# Preincubation of Cervical Swabs in Lim Broth Improves Performance of ICON Rapid Test for Detection of Group B Streptococci

**DOI:** 10.1155/S1064744996000051

**Published:** 1996

**Authors:** Sousan Sayahtaheri Altaie, James Bridges, Darius Loghmanee, Amol Lele, Kenneth R. Kahn

**Affiliations:** 1 School of Medicine and Biomedical Sciences Department of Pediatrics State University of New York Buffalo NY USA; 2 School of Medicine and Biomedical Sciences Department of Obstetrics and Gynecology State University of New York Buffalo NY USA; 3 Children's Hospital of Buffalo Division of Infectious Diseases Buffalo NY USA; 4 Center for Drug Evaluation and Research Division of Anti-Infective Drug Products 5600 Fisher's Lane HFD-520 Rockville MD 20857 USA

## Abstract

*Objective:* The purpose of this study was to determine whether an enrichment method would improve the performance of an enzyme imunoassay test, the ICON Strep B, for detection of group B streptococci (GBS) in vaginoperineal swabs.

*Methods:* The study was done in 3 phases. First, in 250 maternity patients, 2 swabs per patient were tested simultaneously by an overnight selective broth culture method (Lim broth) and the ICON assay. Forty-five (18%) specimens were positive for GBS by culture. The ICON assay detected only 2 (4%) of the positives. Second, in 391 maternity patients, a single swab was cultured as above. However, during the overnight incubation of the Lim broth, 0.5 ml aliquots were removed and tested by ICON assay at 4, 6, 8, 10, and 12 h post-incubation. Seventy-two specimens (18%) were positive by culture. The ICON assay detected 20% of the positives at 4 h, 46% at 6 h, 70% at 8 h, 94% at 10 h, and 100% at 12 h post-incubation. Third, 97 high-risk patients with the diagnosis of preterm labor (PTL)/or preterm premature rupture of the membranes (PPROM) were sampled. Three specimens per patient were obtained: a single swab that was cultured as before and 2 double swabs, of which 1 was tested directly using the ICON test and the other was placed directly in Lim broth and incubated overnight. The aliquots of broth were tested by the ICON assay at 2, 4, 6, and 8 h post-incubation. Twenty-four specimens were positive by culture.

*Results:* The direct ICON test detected only 4 (17%) of the positives. The ICON assay performed on the enriched samples detected 4% of the positives at 2 h, 21% at 4 h, 58% at 6 h, and 100% at 8 h post-incubation.

*Conclusions:* These data indicate that the ICON assay may be used with 100% sensitivity and specificity to detect GBS-colonized high-risk mothers within 8 h if the initial sample size is doubled and the enrichment broth is used in the performance of the ICON assay.


Infectious Diseases in Obstetrics and Gynecology 4:20–24 (1996)
© 1996 Wiley-Liss, Inc.

### Preincubation of Cervical Swabs in Lim Broth Improves Performance of ICON Rapid Test for Detection of Group B Streptococci

Sousan Sayahtaheri Altaie, James Bridges, Darius Loghmanee, Amol Lele, and Kenneth R. Kahn

*School of Medicine and Biomedical Sciences, Departments of Pediatrics (S.S.A.), and Obstetrics and Gynecology (A.L., K.R.K.), State University of New York at Buffalo, and Children's Hospital of Buffalo, Division of Infectious Diseases (S.S.A., J.B., D.L., A.L., K.R.K.), Buffalo, NY*

### Abstract

***Objective:*** The purpose of this study was to determine whether an enrichment method would improve the performance of an enzyme immunoassay test, the ICON Strep B, for detection of group B streptococci (GBS) in vaginoperineal swabs.

***Methods:*** The study was done in 3 phases. First, in 250 maternity patients, 2 swabs per patient were tested simultaneously by an overnight selective broth culture method (Lim broth) and the ICON assay. Forty-five (18%) specimens were positive for GBS by culture. The ICON assay detected only 2 (4%) of the positives. Second, in 391 maternity patients, a single swab was cultured as above. However, during the overnight incubation of the Lim broth, 0.5 ml aliquots were removed and tested by ICON assay at 4, 6, 8, 10, and 12 h post-incubation. Seventy-two specimens (18%) were positive by culture. The ICON assay detected 20% of the positives at 4 h, 46% at 6 h, 70% at 8 h, 94% at 10 h, and 100% at 12 h post-incubation. Third, 97 high-risk patients with the diagnosis of preterm labor (PTL)/or preterm premature rupture of the membranes (PPROM) were sampled. Three specimens per patient were obtained: a single swab that was cultured as before and 2 double swabs, of which 1 was tested directly using the ICON test and the other was placed directly in Lim broth and incubated overnight. The aliquots of broth were tested by the ICON assay at 2, 4, 6, and 8 h post-incubation. Twenty-four specimens were positive by culture.

***Results:*** The direct ICON test detected only 4 (17%) of the positives. The ICON assay performed on the enriched samples detected 4% of the positives at 2 h, 21% at 4 h, 58% at 6 h, and 100% at 8 h post-incubation.

***Conclusions:*** These data indicate that the ICON assay may be used with 100% sensitivity and specificity to detect GBS-colonized high-risk mothers within 8 h if the initial sample size is doubled and the enrichment broth is used in the performance of the ICON assay. © 1996 Wiley-Liss, Inc.

Key words

Vaginal colonization, GBS rapid detection, neonatal sepsis, high-risk pregnancy

Vaginal colonization with group B streptococci (GBS) occurs in 10–30% of normal pregnancies, while neonatal colonization occurs in 40–70% of the infants born to colonized women.[1] Approximately 1–2% of colonized infants ultimately develop early onset neonatal GBS sepsis,[2] a disease with a high mortality rate (22–29%) and much morbidity.[3–5] Up to 15,000 cases of neonatal sepsis each year are caused by GBS.[6] The factors that increase the risk for the development of GBS sepsis include low birth weight and prematurity, prolonged membrane rupture, and maternal factors such as fever in labor

Address correspondence/reprint requests to Dr. Sousan Sayahtaheri Altaie, Center for Drug Evaluation and Research, Division of Anti-Infective Drug Products, Attention: Document Control Room, 5600 Fisher's Lane HFD-520, Rockville, MD 20857.

Received August 24, 1995
Accepted February February 29, 1996

Clinical Study and heavy vaginal colonization. In 1986, Boyer and Gotoff[7] and Morales et al.[8] demonstrated that intrapartum ampicillin prophylaxis in mothers colonized with GBS during the third trimester resulted in a decrease in neonatal colonization and neonatal GBS disease. Recognizing those patients who would most benefit from intrapartum ampicillin prophylaxis, however, remains a major problem. Boyer et al.[9] and Allardice et al.[10] have demonstrated that antepartum cultures may not be predictive of intrapartum colonization status, because only 55–67% of women with positive cultures during the third trimester will have positive cultures at the time of delivery, even in the absence of treatment with antibiotics. In addition, approximately 5–10% of the women with negative antepartum cultures will have positive cultures when they are evaluated intrapartum.[9,10] It is evident that there is a great need for rapid and accurate detection of GBS, especially in those patients whose infants are at high risk for neonatal GBS disease.

The available commercial rapid tests for GBS have sensitivities ranging from 11 to 30% compared with cultures on selective medium.[1,11–13] The current study was undertaken to explore the possibility of using an enrichment broth (to increase the bacterial load) prior to testing the specimen with a rapid assay (ICON) in order to increase the sensitivity of the rapid test and shorten the time for a diagnosis.

### MATERIALS AND METHODS

### Patient Population

During the first phase of the study, from October 1992 to March 1993, 250 maternity patients presenting at Children's Hospital of Buffalo for evaluation of labor or possible rupture of the membranes were enrolled. For the second phase of the study, from July 1993 to February 1994, 391 maternity patients from the same population were enrolled. During the third phase of the study, from April 1994 to April 1995, 97 women admitted to the Children's Hospital with the diagnosis of preterm (<37 weeks gestation) labor (PTL) or preterm premature rupture of the membranes (PPROM) were enrolled.

### Specimen Processing and Culture Method

#### *Phase One*

For the first phase of the study, the 2 vaginoperineal swabs were collected in culturettes containing a rayon-tipped swab and an ampule of modified Stuart's medium (Becton Dickinson, Cockeysville, MD). The first swab with an intact ampule was tested by the ICON assay (Hybritech, Inc., San Diego, CA). Within 4 h from the time of collection, the second swab was plated semiquantitatively on 5% sheep blood agar (BA). The swab then was placed in Lim broth (Todd-Hewitt broth containing 1% yeast extract, 10 μg/ml of colistin, and 15 μg/ml of nalidixic acid), a selective enrichment broth. The BA and the Lim broth were incubated in 5% $CO_2$ and air, respectively, at 36°C overnight. The Lim broth was subcultured onto a BA plate the following day.

#### *Phase Two*

During the second phase of the study, 1 vaginoperineal swab was collected per patient using single-swab culturettes. The specimen was cultured as above except that, during the incubation, 0.5 ml aliquots of the Lim broth were removed at 4, 6, 8, 10, and 12 h post-incubation and frozen at −20°C until needed for ICON testing. The frozen samples were batched and tested once a week. The remaining Lim broth was subcultured to a BA plate at 24 h post-incubation. All media were obtained from BBL (Becton Dickinson). The confirmation of GBS from the cultures was acomplished with one of the following agglutination kits: Streptococci grouping kit (Becton Dickinson) or Streptex (Murex, Diagnostics Limited, Dartford, England).

#### *Phase Three*

In order to increase the initial bacterial load in the specimens, we obtained 3 specimens per patient: 1 single-swab culturette that was cultured as in phase one using BA and 24-h Lim broth; 1 double-swab culturette that was tested directly with the ICON assay; and 1 double-swab culturette that was placed directly into Lim broth and incubated overnight. The aliquots of this Lim broth tube were removed at 2, 4, 6, and 8 h post-incubation and handled as in phase two.

### ICON Assay

#### *Specimen Handling*

To determine the best way by which to process the Lim broth specimens for testing by ICON, we conducted a preliminary study. Fifty known positive (by culture) and 7 known negative Lim broth samples from clinical specimens were tested. Each

TABLE 1. Sensitivity, specificity, and predictive value of direct ICON rapid GBS detection kit in comparison with selective GBS culture[a]

| | Maternity patients[b] (N = 250) | PPROM or PTL[c] (N = 97) | Heavily colonized[d] (N = 30) |
|---|---|---|---|
| Sensitivity (%) | 6 | 17 | 20 |
| Specificity (%) | 100 | 97 | 97 |
| PPV (%) | 100 | 67 | 86 |
| NPV (%) | 83 | 78 | 61 |

[a]PPV, positive predictive value; NPV, negative predictive value.
[b]The ICON assay was performed using a single-swab culturette.
[c]The ICON assay was performed using a double-swab culturette.
[d]The data were analyzed considering only those patients with ≥2+ colonies on the initial BA plate) as positive (30 positives/69 total patients).

sample was divided in 4 1-ml aliquots. The first aliquot was centrifuged for 15 min at 1,500 g, after which the pellet was picked up with a swab (provided with the ICON kit) and tested by the ICON assay. The second and third aliquots were processed as boiled and unboiled urine specimens following the manufacturer's protocol. A swab (provided with the ICON kit) was placed in the fourth aliquot until it was saturated and then tested following the manufacturer's instructions. The results were analyzed and factors such as the amount of labor involved, ease of interpretation (little or no background), sensitivity, and specificity were considered in determining the best way to test the Lim broth by the ICON assay. The results (not shown here) indicated that the best way to test Lim broth for GBS was to saturate a swab with the broth and to test the saturated swab with the ICON assay following the manufacturer's instruction.

### RESULTS

### Phase One

Forty-five (18%) patients were culture positive for GBS. Of these, 29 (64%) had 1+, 7 (16%) had 2+, and 9 (20%) had 3+ to 4+ colonies. Table 1 depicts the value of the ICON assay. As seen, the sensitivity varied from 6 to 20%. In contrast, the specificity of the assay was high at 97–100%. These data suggest that the ICON assay is not valuable as a test performed directly on the initial specimen.

### Phase Two

Seventy-two (18%) patients were culture positive for GBS. Of these, 39 (54%) had 1+, 14 (19%) had 2+, and 19 (26%) had 3+ to 4+ colonies. The ICON assay performed on aliquots of the enrichment broth detected 20% at 4 h, 46% at 6 h, 70% at 8 h, 94% at 10 h, and 100% at 12 h post-enrichment. These data suggest that the ICON shortened the time required for a diagnosis from 48 h by culture to 12 h.

### Phase Three

Twenty-four (25%) patients were culture positive. Of these, 11 (46%) had 1+, 9 (38%) had 2+, and 4 (17%) had 3+ to 4+ colonies. The direct ICON assay detected only 4 positive patients, all of whom had 3+ or 4+ colony counts. As shown in Table 1, when the ICON assay was used to test only the patients with PTL and PPROM, the sensitivity of the direct ICON test improved compared with the ICON assay used to test a mixed maternity population (phase-one patients). However, the specificity of the ICON assay decreased. The direct ICON test was positive in 2 patients with negative cultures. The culture grew only yeast and lactobacilli in 1 patient and coagulase-negative staphylococci, enterococci, lactobacilli, and yeast in the other patient. The ICON assay performed on aliquots of the enrichment broth detected 4% of the positives at 2 h, 21% at 4 h, 58% at 6 h, and 100% at 8 h post-enrichment. As illustrated in Tables 1 and 2, if the ICON assay is used in high-risk populations and if the initial inoculum size is doubled by using double-swabbed culturettes, the sensitivity will increase 3-fold (6% vs. 17%). At 8 h post-enrichment, the sensitivity will reach 100%.

Prior studies of rapid GBS detection systems have shown improved sensitivity only with regard to those patients with heavy vaginal colonization.[1,11–19] Therefore, we analyzed the ICON results from heavily colonized patients in phases one and three,

TABLE 2. Effects of patient population and initial inoculum size on sensitivity of ICON assay performed on specimens enriched over time[a]

| | Sensitivity of ICON assay (%) | |
|---|---|---|
| Time post-enrichment (h) | Maternity patients[b] (N = 391) | PPROM or PTL patients[c] (N = 97) |
| 2 | NP | 4 |
| 4 | 20 | 21 |
| 6 | 46 | 58 |
| 8 | 70 | 100 |
| 10 | 94 | NP |
| 12 | 100 | NP |

[a]NP, not performed.
[b]The ICON assay was performed using a single-swab culturette.
[c]The ICON assay was performed using a double-swab culturette.

defining ≥2+ on the initial BA plate as positive (N = 69). As shown in Table 1, the sensitivity of the ICON assay also improved in our study. The false-negative rate or the percentage of carriers not identified by a rapid assay increases with decreasing colony counts (the lighter the colonization, the greater the false-negative rate). Among patients in phases one and three of the study, only 3% of those with 1+ colonies and 43% of those with 3+ and 4+ colonies were detected by the ICON assay.

### DISCUSSION

Although the first studies to use the ICON assay for the rapid detection of GBS reported higher sensitivities (92% by Park et al.[20] and 33% by Gentry et al.[16]), these results have been criticized for comparing the rapid test with culture results obtained with nonselective medium. A subsequent study of the ICON test in comparison with selective culture revealed a lower sensitivity (11% by Armer et al.[11]). Our finding that the sensitivity of the ICON assay is low is in agreement with Armer et al.[11]

The results with other rapid test methods have proved equally disappointing. Using a different enzyme immunoassay (Equate® assay), Dinsmoor et al.[1] reported a sensitivity of 28%, Skoll et al.[15] 15%, and Greenspoon et al.[14] 33%. All 3 authors concluded that the assay is not sufficiently sensitive in detecting light colonization, which would preclude it from routine use in the clinical setting. Additional studies using latex agglutination tests have resulted in similar findings, with sensitivities ranging from 15 to 30% and specificities from 93 to 100% and negative predictive values of 76–88% in those populations with significant colonization rates.[12–14,18,19,21]

Many studies (including the current study and a previous study of one of the authors[1]) have demonstrated an increased sensitivity of the rapid test when it is used only in heavily colonized women. It remains, however, a matter of discussion as to whether or not the detection of only heavily colonized mothers is adequate.[11,12,23] Isada and Grossman[18] reported that all 9 cases of "clinically significant" perinatal GBS morbidity were identified using a rapid latex agglutination test in a population with a low (4.4%) GBS prevalence. Although heavy maternal colonization increases the risk for neonatal GBS disease, many cases of GBS sepsis have been reported in infants of women who were only lightly colonized.[8,11,22–24] Therapeutic levels of ampicillin can be achieved in fetal serum within hours after a single maternal dose. It has been the basis of antibiotic chemoprophylaxis in a GBS carrier or a patient with an unknown GBS status and PPROM. A rapid antigen test for the detection of GBS in a patient with an unknown carrier status would be of tremendous clinical importance in identifying high-risk patients and instituting chemoprophylaxis against newborn and maternal postpartum sepsis. If the diagnostic test takes longer than 12 h, chemoprophylaxis has to be maintained even in a patient who is later found to be negative.

It is evident that, unless the sensitivity of direct rapid GBS assays is improved to detect even light colonization, their use in chemoprophylaxis protocols is to be discouraged. If a "good" rapid test for direct detection of GBS is one that can be performed within 2 h of specimen collection with >95% sensitivity, the direct ICON assay does not seem to have a place in chemoprophylaxis protocols to identify patients at high risk for neonatal sepsis. In our study, after determining the baseline sensitivity of the ICON assay (phase one), we took 2 approaches to increase the sensitivity of the assay. First, we utilized an enrichment broth method prior to testing (phase two). In a mixed maternity population, we were able to detect GBS in 94% and 100% of the colonized patients 10 and 12 h post-enrichment, respectively. Second, we increased the starting inoculum size by sampling double swabs (phase three). In a high-risk population, we were able to detect GBS in 100% of the colonized patients 8 h post-enrichment.

Our findings suggest that the double-swab and enrichment technique may be used in conjunction with the ICON assay to reliably replace the culture method and shorten the diagnostic time from 48 h to <12 h. This approach may be used to continue antibiotic therapy in colonized patients, discontinue therapy in noncolonized patients, or appropriately treat at-risk patients.

### REFERENCES

1. Dinsmoor MJ, Dalton HP, Thomas CC, et al.: Comparison of culture and rapid enzyme immunoassay for detection of group B streptococcus in high-risk pregnancies. Infect Dis Obstet Gynecol 2:115–119, 1994.
2. Wilkinson HW: Group B streptococcal infection in humans. Annu Rev Microbiol 32:41–57, 1978.
3. De-Lovois J, Blackburn J, Hurley R, et al.: Infantile meningitis in England and Wales: A two year study. Arch Dis Child 66(5):603–607, 1991.
4. Philip AG: The changing face of neonatal infection: Experience at a regional medical center. Pediatr Infect Dis J 13(12):1098–1102, 1994.
5. Hristeva L, Booy R, Bowler I, et al.: Prospective surveillance of neonatal meningitis. Arch Dis Child 69:14–18, 1993.
6. Lim DV, Morales WL, Walsh AF, Kazanis D: Reduction of morbidity and mortality rates for neonatal group B streptococcal disease through early diagnosis and chemoprophylaxis. J Clin Microbiol 23:489–492, 1986.
7. Boyer KM, Gotoff SP: Prevention of early-onset neonatal group B streptococcal disease with selective intrapartum chemoprophylaxis. N Engl J Med 314:1655–1699, 1986.
8. Morales WJ, Lim DV, Walsh AF: Prevention of neonatal group B streptococcal sepsis by use of a rapid screening test and selective intrapartum prophylaxis. Am J Obstet Gynecol 155:979–983, 1986.
9. Boyer KM, Gadzala CA, Kelly PD, Burd LI, Gotoff SP: Selective intrapartum chemoprophylaxis of neonatal group B streptococcal early-onset disease. II. Predictive value of prenatal cultures. J Infect Dis 148:802–809, 1983.
10. Allardice JG, Baskett TF, Seshia MMK, et al.: Perinatal group B streptococcal colonization and infection. Am J Obstet Gynecol 142:617–620, 1982.
11. Armer T, Clark P, Duff P, Saravanos K: Rapid intrapartum detection of group B streptococcal colonization with an enzyme immunoassay. Am J Obstet Gynecol 168:39–43, 1993.
12. Clark P, Armert T, Duff P, Davidson K: Assessment of a rapid latex agglutination test for group B streptococcal colonization of the genital tract. Obstet Gynecol 79:358–363, 1992.
13. Kontnick CM, Edberg SC: Direct detection of group B streptococci from vaginal specimens compared with quantitative culture. J Clin Microbiol 28:336–339, 1990.
14. Greenspoon JS, Fishman A, Wilcox JG, Greenspoon RL, Lewis W: Comparison of culture of group B streptococci versus enzyme immunoassay and latex agglutination rapid tests: Results of 250 patients during labor. Obstet Gynecol 70:97–100, 1991.
15. Skoll MA, Mercer BM, Baselski V, Gray JP, Ryan G, Sibai BM: Evaluation of two rapid group B streptococcal antigen tests in labor and delivery patients. Obstet Gynecol 77:332–336, 1991.
16. Gentry YM, Hillier SL, Eschenbach DA: Evaluation of rapid enzyme immunoassay test for detection of group B streptococcus. Obstet Gynecol 78:387–401, 1991.
17. Granato PA, Petosa MT: Evaluation of a rapid screening test for detecting group B streptococci in pregnant women. J Clin Microbiol 29:1536–1538, 1991.
18. Isada NB, Grossman JH: A rapid screening test for the diagnosis of endocervical group B streptococci in pregnancy: Microbiologic results and clinical outcome. Obstet Gynecol 70:139–141, 1987.
19. Green M, Dashefsky B, Wald ER, Laifer S, Harger J, Guthrie R: Comparison of two antigen assays for rapid intrapartum detection of vaginal group B streptococcal colonization. J Clin Microbiol 31:78–82, 1993.
20. Park CH, Hixon DL, Spencer ML, et al.: The ICON strep B immunoassay for rapid detection of group B streptococcal antigen. Lab Med 23:543–546, 1992.
21. Brady K, Duff P, Schilhag JC, Herd M: Reliability of a rapid latex fixation test for detecting group B streptococci in the genital tract of parturients at term. Obstet Gynecol 73:678–681, 1989.
22. Towers CV, Garite TJ, Friedman WW, Pircon RA, Nageotte MP: Comparison of a rapid enzyme-linked immunosorbent assay test and the gram stain for detection of group B streptococcus in high-risk antepartum patients. Am J Obstet Gynecol 163:965–967, 1990.
23. Yancey MK, Armer T, Clark P, Duff P: Assessment of rapid identification tests for genital carriage of group B streptococci. Obstet Gynecol 80:1038–1047, 1992.
24. Morales WJ, Lim D: Reduction of group B streptococcal maternal and neonatal infections in preterm pregnancies with premature rupture of membranes through a rapid identification test. Am J Obstet Gynecol 157:13–16, 1987.
